# Chitosan‐enhanced sensitivity of mercaptoundecanoic acid (MUA)‐ capped gold nanorod based localized surface plasmon resonance (LSPR) biosensor for detection of alpha‐synuclein oligomer biomarker in parkinson’s disease

**DOI:** 10.1002/bab.2653

**Published:** 2024-09-03

**Authors:** Begum Balkan Apaydın, Tugay Çamoğlu, Zeliha Cansu Canbek Özdil, Duygu Gezen‐Ak, Duygu Ege, Murat Gülsoy

**Affiliations:** ^1^ Institute of Biomedical Engineering Boğaziçi University Istanbul Turkey; ^2^ Department of Neuroscience, Institute of Neurological Sciences Istanbul University‐Cerrahpaşa Istanbul Turkey; ^3^ Materials Science and Nanotechnology Engineering Yeditepe University Istanbul Turkey

**Keywords:** chitosan, fiber optic biosensor, gold nanorod, oligomer alpha‐synuclein, LSPR, Parkinson's disease, alpha‐synuclein oligomers

## Abstract

Alpha‐synuclein oligomers play a crucial role in the early diagnosis of Parkinson's disease (PD). In this study, a mercaptoundecanoic acid (MUA)‐capped gold nanorod (GNR)‐coated and chitosan (CH)‐immobilized fiber optic probe has shown considerable sensitivity of its detection. The proposed U‐shaped fiber optic biosensor based on localized surface plasmon resonance (LSPR) was applied to detect α‐syn oligomer (OA) biomarker. By analyzing OA concentrations, the biosensor achieved a limit of detection of (LOD) 11 pM within the concentration range of 10–100 pM and the sensitivity value was found as 502.69 Δλ/RIU. Upon analysis of the CV% (coefficient of variation) and accuracy/recovery values, it is revealed that the sensor successfully fulfilled the criteria for success, displaying accuracy/recovery values within the range of 80%–120% and CV% values below 20%. This sensor presents significant advantages, including high sensitivity, specificity, and ability to detect very low concentrations of OA. In conclusion, the suggested U‐shaped fiber optic biosensor has the potential to be valuable in the early detection of PD from a clinical perspective.

**Abbreviations**
CH: chitosan
GNRs: gold nanorods
LOD: limit of detection
LSPR: localized surface plasmon resonance
L‐SPR: longitudinal surface plasmon resonance
MUA: mercaptoundecanoic acid
OA: α‐syn oligomer
PD: Parkinson's disease
T‐SPR: transverse surface plasmon resonance

## 1 INTRODUCTION

The human brain holds a significant quantity of α‐syn which is a 140 amino acid protein primarily located in presynaptic synapses.^1^, ^2^, ^3^ The exact function of α‐syn is unclear, but it may influence the SNARE complex responsible for neurotransmitter release. Parkinson's disease, associated with α‐syn buildup, has an increased risk linked to α‐syn gene locus triplication, leading to higher α‐syn levels.^1^, ^4^, ^5^ Moreover, specific α‐syn variations, such as A53T and A30P, have been implicated in PD,^6^, ^7^ hindering the breakdown of α‐syn, leading to its accumulation.^8^ Monomeric α‐syn change into oligomers when the equilibrium between α‐syn production and clearance is disrupted.^9^

Recent evidence emphasizes the crucial role of oligomeric α‐syn, detectable in cerebrospinal fluid (CSF), blood, and saliva, in the onset of PD.^10^, ^11^, ^12^, ^13^, ^14^ Studies have identified α‐syn oligomers (OAs) in the blood and CSF of PD patients, hinting at their potential use as diagnostic biomarkers.^15^, ^16^, ^17^, ^18^ ELISA and single‐molecule immunoassays utilizing highly specific antibody pairs have been employed to detect OAs.^19^, ^20^, ^21^, ^22^ Yet, developing antibodies specific to pathogenic α‐syn assemblies, including oligomers, remain challenging as current aggregate‐specific antibodies cannot distinguish between oligomers and fibrils.^23^

Circular dichroism,^24^ Fourier transform infrared spectroscopy (FTIR),^25^ Western blot,^26^ ELISA,^27^ and thioflavin T (ThT) fluorescence^28^ are among the techniques used to detect OAs. However, these methods have limitations such as high cost, slow response time, and the need for bulky instruments. Hence, the oligomer‐based α‐syn protein detection technique stands out due to its simplicity, high sensitivity, and specificity. Nevertheless, longitudinal surface plasmon resonance (LSPR)‐based fiber optic biosensors exhibit high precision, specificity, and sensitivity in protein detection.^29^ Additionally, these biosensors offer rapid response times, portability, and suitability for on‐site applications.^30^ Therefore, LSPR‐based fiber optic biosensors hold promise as effective alternatives for α‐syn detection.

Fiber optic biosensors operate based on the coherent interaction between incoming photons and metal nanoparticles. Among the commonly used metal nanoparticles in LSPR applications are GNRs, distinguished by two absorption bands: the longitudinal surface plasmon resonance (L‐SPR) and the transverse surface plasmon resonance (T‐SPR). L‐SPR and T‐SPR represent the electron oscillation along the longer and shorter axes of GNRs, respectively. The sensitivity of GNRs to changes in size and the surrounding medium's refractive index (RI) varies between L‐SPR and T‐SPR. TSPR demonstrates lower sensitivity to these alterations, while L‐SPR exhibits a shift to longer wavelengths (redshift) in response to an increased aspect ratio of GNRs, displaying heightened sensitivity to RI changes.^31^

The characteristics of LSPR heavily rely on nanoparticle size, shape, dielectric properties, and the nature of the surrounding environment, impacting the electron charge density on the particle surface.^33^, ^34^ LSPR is highly responsive to changes in the RI within the local environment. In numerous sensing methodologies, alterations in peak wavelength or absorbance in the absorption spectrum of MNPs are employed as indicators of LSPR sensor response.

Another crucial factor influencing the effectiveness of LSPR‐based fiber optic biosensors is the design of the FO probe. Studies have indicated that U‐shaped FO probes can significantly enhance sensitivity, especially for biomarkers with low concentrations, like oligomeric α‐syn, due to their heightened RI sensitivity.^31^

11‐Mercaptoundecanoic acid (MUA) is used to form a self‐assembled monolayer on the surface, thereby enhancing sensor performance.^32^ This enhancement encompasses the improvement of target molecule binding to the sensor surface, an increase in detection sensitivity, and the establishment of stability in the sensor surface.^33^, ^34^ MUA was selected for the surface functionalization of nanoparticles due to its nontoxic properties.^35^ The interaction between MUA and GNRs is attributed to the affinity of thiol groups for the gold nanoparticles.^36^ Consequently, MUA emerges as the most suitable molecule for establishing a stable surface within the scope of this study.

Chitosan (CH), a carbohydrate polymer, has emerged as a valuable transducer owing to its robust mechanical properties, strong adhesive behavior, biocompatibility, biodegradability, high availability, and sustainability.^37^ As a polysaccharide, CH can interact with amyloid molecules through its glucosamine groups, positioning it as a promising candidate for sensor applications.^38^, ^39^

**FIGURE 1:**
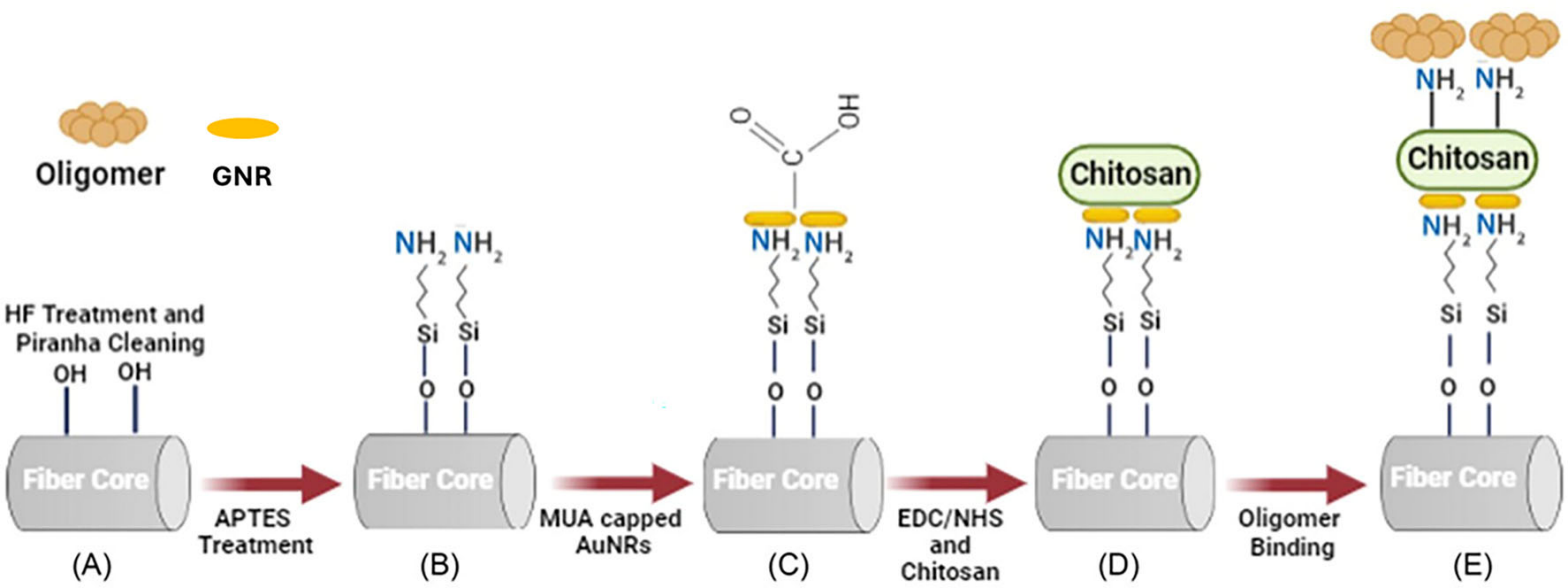
Fabrication of the sensor probe. Functionalization respectively, (A) Cleaning fiber probe by using Piranha Solution sulfuric acid and hydrogen peroxide (4:1), (B) APTES treatment, (C) MUA‐capped GNRs coating, (D) EDC/NHS treatment and chitosan coating, (E) Oligomer form of α‐syn detection.

In this study, U‐shaped LSPR based fiber optic biosensor probe was specifically tailored for detecting α‐syn oligomers MUA‐capped GNRs were immobilized onto the fiber‐optic probe's core and bio‐functionalized with CH to create the sensing region on the fiber (Figure 1). The outcomes of this study demonstrated that the biosensor developed using a CH‐coated sensor probe could detect α‐syn oligomers with a low LOD and high selectivity.

## 2 MATERIALS AND METHODS

### 2.1 Reagents and materials

All reagents required for this work were acquired commercially and used without further purification. These included cetyltrimethylammonium bromide (CTAB 99%) and tetra chloroauric(III) acid (extra pure) (HauCI_4_. 3H_2_O) from Sigma‐Aldrich, along with silver nitrate (extra pure) (AgNO_3_,extra pure),, sodium borohydride (98%) (NaBH_4_), trisodium citrate (Na_3_C_6_H_5_O_7_) , high‐molecular‐weight CH (HW), *N*‐(3‐dimethylaminopropyl)‐*N*′‐ethylcarbodiimide hydrochloride (EDC), *N*‐hydroxysuccinimide (NHS), l‐ascorbic acid, 11 MUA, and 3‐aminopropyltriethoxysilane (APTES) purchased from Sigma‐Aldrich. Additional items obtained were absolute ethanol, methanol, sodium hydroxide (NaOH), phosphate buffer saline (PBS) tablets, and hydrochloric acid from Merck Chemicals. Deionized (DI) water was produced using a water purification system.

### 2.2 The crafting and cleansing of the U‐formed fiber optic detection device

A fiber optic probe was constructed using an optical fiber featuring a 400 µm diameter core and a rigid polymer cladding with 425 μm diameter (purchased from Thorlabs). The fiber underwent polishing on both ends, and hydrofluoric acid (HF) (48%) was utilized to eliminate the cladding section of the fiber. To create the U‐shape configuration for the probe, the etched area was subjected to a butane flame within the 2 cm region's center and then manually bent to achieve the desired curvature. Following this, the fiber probe was immersed in a fresh piranha solution (comprising H_2_O_2_:H_2_SO_4_ in a 1:4 ratio) at 60°C for approximately 20 min to eradicate all organic and inorganic impurities. After this treatment, the probe underwent multiple rinses with DI water to eliminate any residual acidic substances. Afterward, the fiber was dried using a stream of nitrogen and then heated in an oven at a temperature of 100°C for 30 min to generate hydroxyl groups on the glass surface.

### 2.3 Functionalization of fiber optic sensor probe

The sensor probe, treated with piranha solution, was then immersed in a 1% (v/v) APTES solution overnight. Following rinsing with ethanol, the probe was heated to 80°C in a hot air oven for 2 h to create a strong and consistent silane layer. Afterward, the probes underwent a cleaning process involving ethanol and DI water. The probes were dried using nitrogen gas and then subjected to heat at 110°C in an oven for 1 h. After establishing the amino‐silanized layer on the sensor probe, the probe was dipped into an MUA‐capped GNR solution for 24 h. Further treatment involved exposing them to an EDC–NHS solution (400 mM:100 mM) for 3 h at room temperature (RT). A solution containing 0.015 g of CH dissolved in 12.5 µL acetic acid and 1.2 mL DI water was vortexed for 3 h to achieve a uniform solution. The probes treated with EDC–NHS were placed in a CH solution for 24 h. These probes, coated with GNRs and functionalized with CH, were used to detect oligomers. The CH coating durations were implemented for 48, 36, and 24 h, respectively, and the CH thicknesses on the fiber probe were observed to be 2.26 µm, 120 nm, and 75 nm.

### 2.4 Synthesis of GNRs

#### 2.4.1 Synthesis of GNR seeds

CTAB‐coated seeds were produced through a modified method derived from the seed‐mediated technique described by Nikoobakht and El‐Sayed.^40^ Essentially, the nanoparticles were formed by combining a freshly made ice‐cold NaBH_4_ solution (0.1 mL, 0.0264 M) with a solution containing HAuCl_4_ (0.025 mL, 0.05 M) and CTAB (4.7 mL, 0.1 M). This resultant mixture underwent vigorous stirring for 2 min while kept at a temperature of 28°C. Following its preparation, the solution, exhibiting a faint brown tint, was employed for subsequent experiments an hour later.

#### 2.4.2 Growth of seeds

After the seeds are made, they are turned into GNRs in the next stage of the manufacturing process. To create a growth solution, a specific sequence of substances is added to a 10 mL aqueous solution of 100 mM CTAB. This includes 530 µL of 10 mM HauCl_4_·3H_2_O, 140 µL of 10 mM·AgNO_3_, 75 µL of 100 mM l‐ascorbic acid, and 2 µL of 5 M HCl (37%). Once all of these chemicals are combined, 60 µL of the previously prepared seed solution is quickly added to the growth solution, whereas it is stirred continuously at 30°C. The solution is then left untouched overnight at 30°C.

#### 2.4.3 MUA‐capped GNRs

A technique based on pH was used to remove CTAB completely from the surface of the synthesized GNRs. The process involved the following steps: First, any excess CTAB from GNR solutions with different aspect ratios was removed by centrifugation. Then, the GNRs were suspended in DI water, and a 20 mM MUA aqueous solution was prepared by dissolving 44 mg of MUA in 10 mL of DI water. The MUA solution was sonicated, and a NaOH (0.2 M) solution was added to it while stirring and undergoing periodic sonication until the MUA was dissolved. For the replacement of CTAB present on the GNRs’ surface, the MUA solution was stirred for varying durations (2, 4, 6, and 24 h) in the presence of purified GNR solutions.

### 2.5 Oligomer alpha‐synuclein formation

One milligram of recombinant alpha‐synuclein (Millipore, AG938‐1MG) was dissolved in a buffer containing 20 mM Tris–HCl (pH 7.5), 0.1 M NaCl, and 1 mM MgCl_2_, resulting in a concentration of 1 mg/mL. To eliminate potential fibrils, the proteins were centrifuged at 20,000 *g* for 20 min at 4°C, and the resulting supernatant was stored at −80°C until further use. The oligomerization process followed a previously described method with some modifications.^41^ The proteins were thawed at RT and incubated for 16 h at 37°C without agitation, followed by an additional 12‐h incubation at 56°C. Subsequently, the reaction samples were centrifuged at 20,000 *g* for 20 min at 4°C. The confirmation of oligomerization was achieved through Western blot analysis, ThT testing, and electron microscopy. Monomeric proteins were utilized as a control in these experiments. For the ThT test, in the final reaction, 4.5 µM concentrations of monomeric and oligomeric alpha‐synucleins were combined with 20 µM ThT (Sigma‐Aldrich, T3516‐5G) in PBS. The samples were placed in black wall clear bottom 96‐well plates (Corning, 3904) and incubated for 1 h at 37°C with continuous shaking at 400 rpm. The Biotek Synergy HTX Multi‐Mode Reader was used to measure fluorescence intensity with excitation set at 440 nm and emission at 485 nm. The fluorescence intensities between the oligomers and monomers were compared.

#### 2.5.1 Western blot analysis

Monomers and oligomers were mixed with a 4× sample buffer (Thermo Fisher, B0007) containing 10% beta‐mercaptoethanol and boiled for 5 min at 100°C. Following this treatment, the samples were loaded into 4%–12% polyacrylamide gels (Thermo Fisher, NW04120BOX) and transferred onto PVDF membranes (Thermo Fisher, PB5310) using a Trans‐Blot Turbo system (Biorad, 1704150). The membranes underwent blocking with 5% skim milk for 1 h at RT. They were then incubated overnight with an anti‐alpha‐synuclein antibody (Thermo Fisher, 328100) at a dilution ratio of 1/375, followed by a 2‐h incubation at RT with a secondary antibody (Abcam, ab97040) at a dilution ratio of 1/3000. Subsequently, the membranes were treated with a chemiluminescent substrate (Thermo Fisher, 34580), and the resulting signals were captured using the Microchemi 4.2 imaging system (DNR).

### 2.6 Characterization methods

In order to investigate how CH attaches to specially made fiber probes, multiple characterization techniques were employed. Fourier‐transform infrared spectroscopy (FTIR) spectra were analyzed using a Nicolet 6700 Thermo FT‐IR Spectrometer within the 7800 to 350 cm^−1^ range to study the chemical bonding and functional groups involved. Raman spectroscopy measurements were conducted with Nd:YAG excitation and 0.3 cm^−1^ resolution in the spectral range of 200 to 2200 cm^−1^, employing the Renishaw in Via Reflex Raman spectrometer to complement the FTIR data with information on vibrational modes. To analyze the optical characteristics of the synthesized gold nanorods, UV‐vis absorption spectroscopy was performed using a Varian Cary 100 Bio UV‐Vis Spectrophotometer, recording the absorption spectrum in the range of 190‐900 nm with a quartz cuvette of 10 mm path length. To observe the probe's surface topography, scanning electron microscopy (SEM) was utilized. For this, a uniform layer of gold with a thickness of 5 nm was prepared to enhance conductivity, and images were captured using a Philips XL30 ESEMFEG/EDAX instrument at 20 kV in a specific working mode. Transmission electron microscopy (TEM) was applied to produce high‐resolution micrographs of oligomer and monomer protein species, as well as GNR particles. For TEM analysis, samples were placed on copper grids coated with formvar/carbon films and stained with a 1% aqueous uranyl acetate solution. The micrographs were obtained using a JEM 2100 Plus TEM equipped with a Gatan US4000 CCD.

### 2.7 Concentration analysis

The sensor probe was exposed to oligomer α‐synuclein concentrations (10, 20, 40, 100 pM) at room temperature, washed with PBS, and blocked with Bovine Serum Albumin (BSA) to prevent non‐specific binding. Detection performance was evaluated for CV% in Eq. (1) and accuracy/recovery values in Eq. (2)

(1) CV%=SD/mean×100

(2) $$\begin{array}{ccc} \text{Accuracy}/\mathrm{recovery}\% & = & (\mathrm{calculated}\;\mathrm{mean})/\\ & & \left(\mathrm{theoretical}\;\mathrm{mean}\right) \end{array}$$

## 3 RESULTS AND DISCUSSION

In this study, CH was coated on MUA‐capped fiber probes to improve the sensitivity of the sensors. Various methods, including probe functionalization, nanorod production, and protein formation, were used to characterize the sensor produced and reveal its sensitivity. The results obtained are discussed below.

### 3.1 Gold Nanorod and MUA‐capped Gold Nanorod analysis

The U‐shaped LSPR‐based fiber optic probe fabrication strategy is displayed in Figure 1.

**FIGURE 2:**
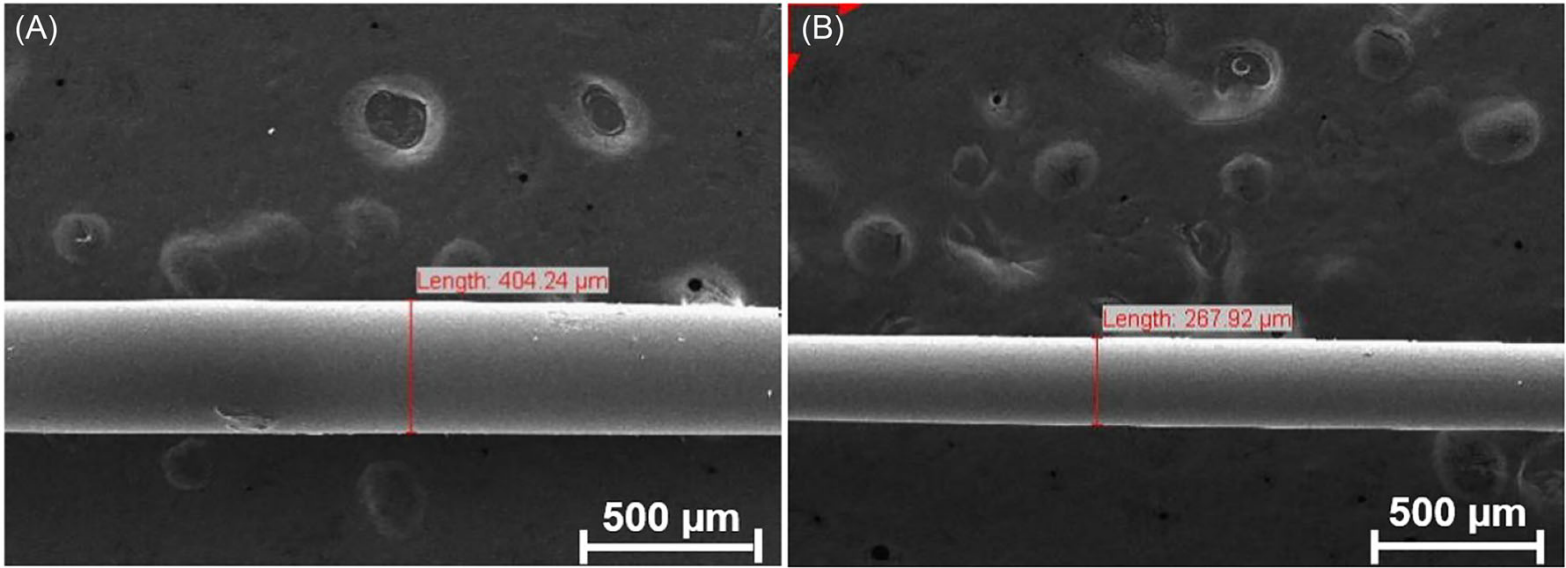
Scanning electron microscopy imaging of the fiber probe (A) before HF etching and (B) after HF etching.

U‐shaped multimode fiber probe was fabricated by chemical etching with hydrofluoric acid of the cladding region.^42^, ^43^ The fiber immersed in a 48% HF solution, ensuring uniform coverage. The HF acid diffuses slowly into the silica lattice, potentially altering the mass of fiber.^44^ The thickness of the fiber, which initially was 404.24 µm, decreased to 267.92 µm after chemical etching, as observed in the SEM image in Figure 2. The reduction in the fiber core diameter to 267.92 µm is a direct consequence of the etching process and the etching process has increased the surface area of the sensor. By decreasing the fiber thickness, the interaction of the evanescent field with the surrounding medium increased, thereby improving the sensitivity of the sensor to changes in the refractive index (RI)^45^, ^46^, ^47^

**FIGURE 3:**
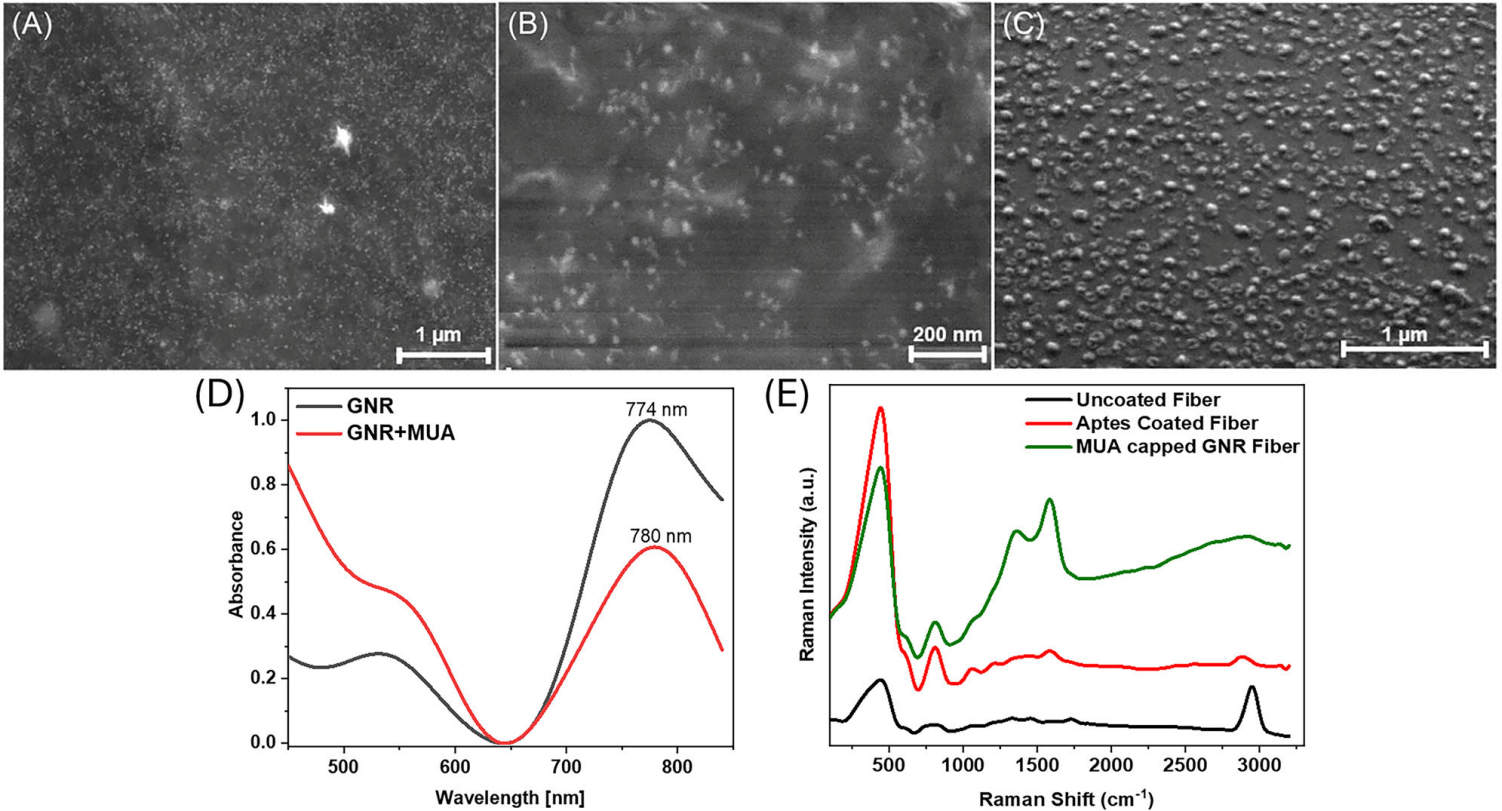
SEM images of fiber probe surfaces with (A,B) non‐aggregated MUA‐capped GNRs with pH 10 (C) aggregated MUA‐capped GNRs with pH 4 and pH 5, (D) Normalized UV‐visible spectra of GNR (black line) and MUA‐capped GNR (red line) occurrence were observed after 24 h stirring. After GNRs were coated with MUA, the wavelength shift was monitored from 774 nm to 780. (E) Raman spectra of sensor probe for bare fiber (dark green), APTES coated (light green), and MUA‐coated fiber (red).

Accordingly, GNRs and MUA‐capped GNRs were prepared through multiple steps. The hydroxylated working probe was incubated in MUA‐capped GNR overnight to construct the immobilization matrix. The synthesized MUA‐capped GNRs were immobilized on the sensor probe surface after being treated with APTES solution and the morphological changes were observed by using SEM and Raman in Figure 3.

Due to instability of CTAB bilayer, the passivation of the nanorod surface by MUA ligand takes place.^48^ Parameters like pH value and stirring time are essential for synthesizing MUA‐capped GNRs. SEM images show demonstrating GNR–MUA complexes’ dispersion. This shows no substantial changes in their morphology compared to the initially synthesized MUA‐capped GNRs under pH 10. The stability of MUA‐capped GNR prepared in a basic environment and its homogeneous attainments have also been observed in other studies.^49^, ^50^ As shown in Figure 3A,B, it is evident that MUA‐capped GNRs (stirred for 24 h at pH 10) on the sensor probe surface maintained homogeneous distribution. The nanoparticle size was shown in SEM images, indicating that particle sizes are within an acceptable range (Figure S1). At lower pH values (stirred 4 h at pH 5 and), the increase of interactions leads to aggregation of MUA, which can be observed in Figure 3C.

The absorption spectra depicted in Figure 3D showcase distinct plasmon bands for both GNRs and MUA‐capped GNR solutions. Notably, the transverse plasmon wavelength (TPW) appears near 530 nm, whereas the longitudinal plasmon wavelength (LPW) peaks around 774 nm for GNRs. The MUA–GNR spectrum showed a redshift from 774 to 780 nm in the longitudinal surface plasmon peak because of surface modification, and the TPW showed from 530 to 540 nm. In the ligand exchange process, thiols attach to gold surface through Au–S bonds, reducing the number of free electrons of GNR. Increasing electron density results in a higher Surface Plasmon Resonance (SPR) frequency; thus, the SPR shifts toward the red end of the spectrum when the electron density is decreased.^51^, ^52^ The longitudinal surface plasmon peak shift is attributed to alterations in the RI at the GNRs’ surface, resulting from the ligand exchange. Previous research reveals that LPW is more sensitive to changes in the RI within the surrounding environment of GNRs than TPW.^53^, ^54^ These findings substantiate prior reports, affirming the heightened sensitivity of GNRs with increased aspect ratios. Figure 3E  shows Raman analysis which was conducted to evaluate the bonding of GNRs coating on U‐shaped probes, both before and after the layer with MUA‐capped GNRs. The surface functionalization process on the fiber surfaces was achieved via a self‐assembly method utilizing APTES monolayer formation. This process was initiated by the piranha treatment, which introduced abundant surface hydroxyl groups (–OH) on the fiber samples through a hydroxylation process. A distinctive peak between 2900 and 3010 cm^−1^ on the uncoated fiber surface indicates the existence of –OH bonds on the surface. Subsequently, during the silanization step, APTES self‐assembled monolayers were formed on the fiber surface, showcasing various atomic bands in the vertical direction. Notably, specific vibrations were observed at 327 and 1605 cm^−1^, as indicated.^55^ The thiol (SH) groups present strong adherence to the GNR surface by forming a covalent metal–sulfur bond, thereby creating a well‐ordered, self‐assembled monolayer. The process involved the addition of an aqueous GNR particle suspension to a solution containing this alkane and ethanol. Various vibronic modes of the hydroxyl groups (around 2900 cm^−1^) and the end groups of the thiol molecule (about 750 cm^−1^) were observed in the Raman spectrum.^56^

### 3.2 Sensor probe analysis

**FIGURE 4:**
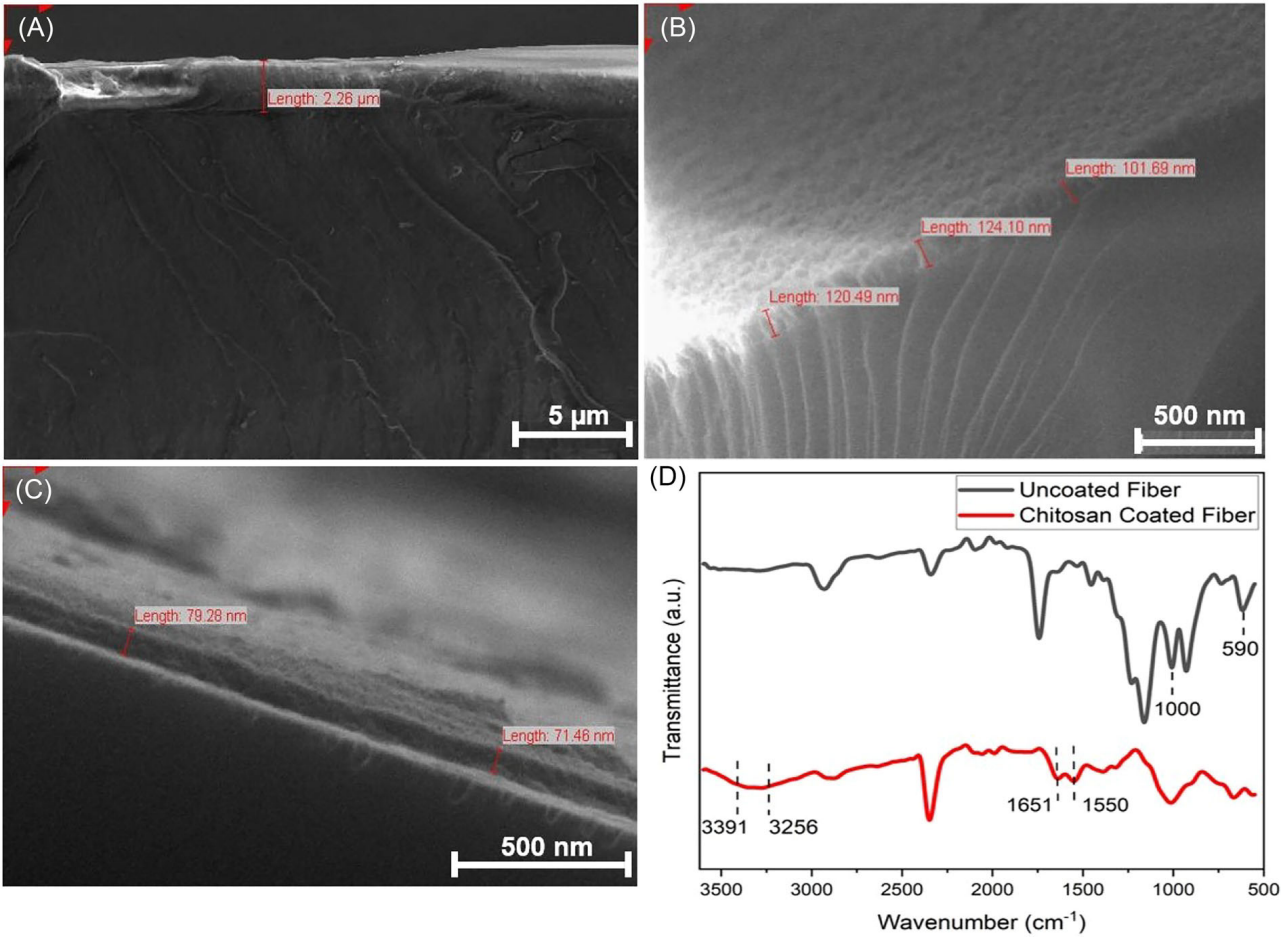
Cross‐sectional scanning electron microscopy (SEM) images of the probe showing chitosan film thicknesses after (A) 48 hours (B) 36 hours, and (C) 24 hours (D) FTIR spectra of sensor probe immobilized with gold nanorods: bare fiber probe (black line) chitosan coated fiber probe (red line) in the 500 cm^‐1^ and 4000 cm^‐1^ spectral ranges. Chitosan chemical composition is presented through spectral ranges between 3256 cm^‐1^ and 3391 cm^‐1^. Other vibrations obtained at 1550 cm^−1^ and 1651 cm^−1^ represent NH2 bending/Amide I band & C=O, respectively.

The chemically treated MUA‐capped GNRs immobilized probe underwent a process to establish a stable CH film. Figure 4 presents SEM images of the thickness of CH‐coated on the fiber probe and the analysis conducted using FTIR. The resulting CH film thicknesses were measured at 2.26 µm after 48 h (Figure 4A), 122 ± 3 nm after 36 h (Figure 4B), and 75 ± 4 nm after 24 h (Figure 4C) by using SEM. The sensor probes were incubated in CH solution for 48, 36, and 24 h, respectively. FTIR spectroscopy was utilized to illustrate the binding of CH onto the sensor probe. Figure 4D displays the FTIR spectra of the fiber probe surfaces, depicting the spectra before (black line) and after (red line) the functionalization with CH. Through FTIR spectroscopy analysis, the chemical composition of the fiber probe surfaces before and after CH modification was characterized. Upon CH functionalization, the FTIR spectrum of the fiber optic sensor probe region exhibited distinctive features. A notable transmission dip was observed around 3256 and 3391 cm^−1^, which signifies the overlapping O–H and N–H stretching vibrations.^57^ Additionally, vibrations observed at 1550 and 1651 cm^−1^ correspond to NH_2_ bending/amide I band and C═O, respectively. An important observation was the absence of the N–H stretching at 3256 cm^−1^ in the spectrum obtained for optical sensor probes solely immobilized with GNRs. This absence confirms the cross‐linking of CH, further confirming its successful binding and presence on the fiber probe surface.^58^

### 3.3 Oligomerization of alpha‐synuclein

**FIGURE 5:**
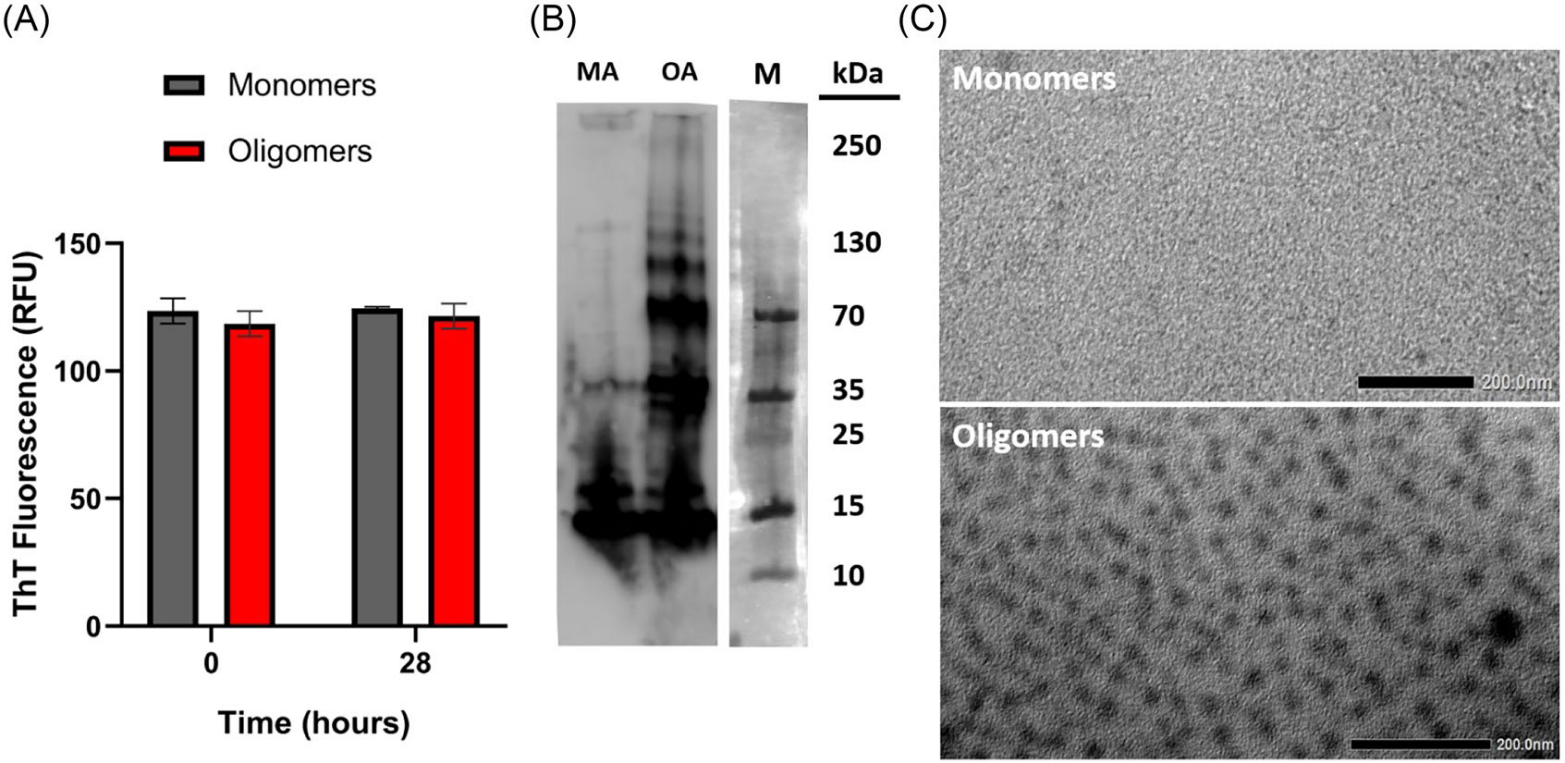
Verification of oligomerization. (A) The graph displays the fluorescent intensity of monomers and oligomers at the initial (T0) and final (T28) stages of the reaction. The samples exhibited similar and non‐increasing fluorescent intensities, indicating that oligomerization did not induce fibrillization. (B) A Western blot comparison between oligomerized samples and monomers revealed the presence of higher molecular weight structures in oligomerized samples. The marker of this membrane was visualized separately using colorimetric imaging. (C) Transmission Electron microscopy (TEM) analysis demonstrated that oligomerized samples (below) formed larger structures than monomers (above), supporting the findings from the ThT test and Western blot. (MA: monomeric alpha‐synuclein, OA: oligomeric alpha‐synuclein, M: marker).

Oligomerization was performed without agitation to prevent fibrillization. We used Western blot, ThT test, and electron microscopy to show the protocol's success. The ThT test showed that there are no fibrils in monomeric and oligomeric samples in Figure 5. ThT is an amyloid‐binding dye that initiates strong fluorescent signals when bound to amyloid fibrils. According to the ThT test, oligomers showed the same fluorescent signal intensity as monomers and the test buffer that contains only ThT. Therefore, it has been demonstrated that oligomerization did not cause fibrillation. Otherwise, Western blot and electron microscopy analysis verified the oligomerization by molecular weight. Western blot analysis showed that the oligomerization samples had higher molecular weight complexes besides monomers. The findings of electron microscopy supported the ThT test and Western blot analysis.

### 3.4 Refractive index sensitivity

**FIGURE 6:**
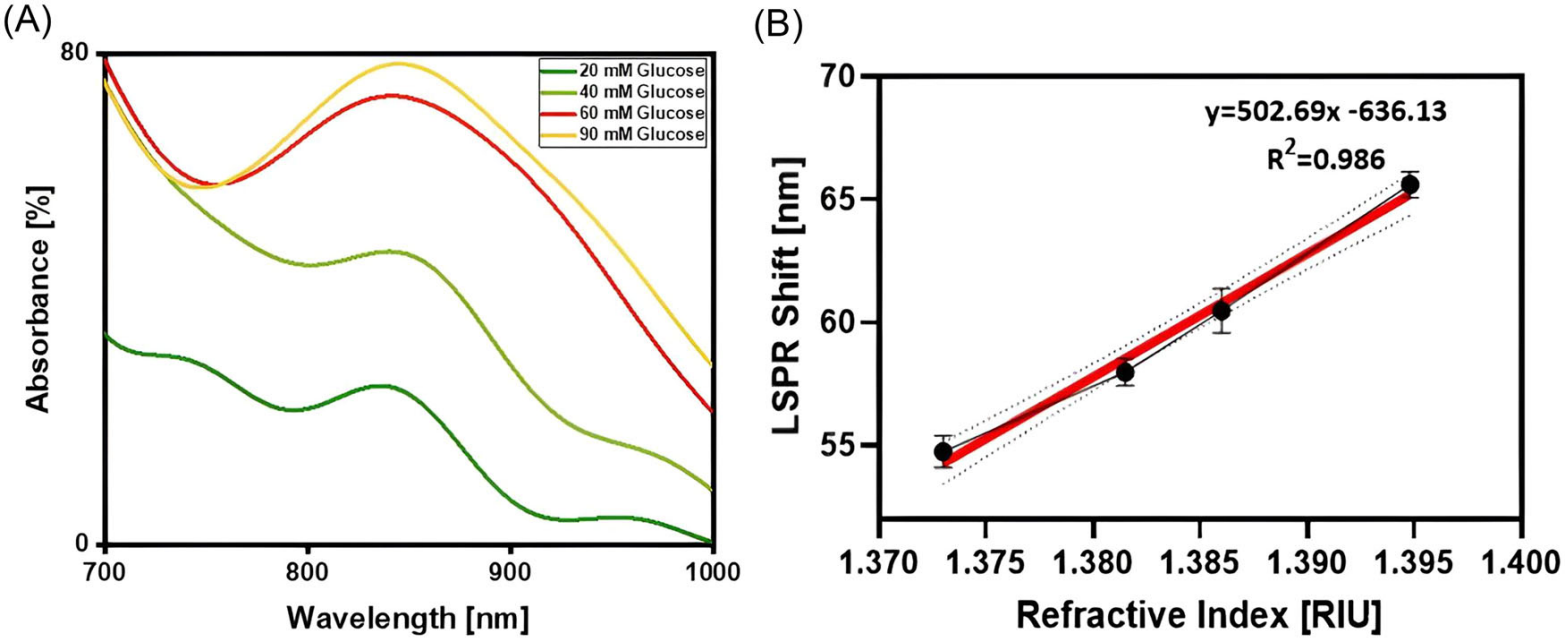
These graphics depict a nanostructured sensor's light absorption and scattering properties for measuring variations in refractive index. A) absorbance changes for MUA‐capped GNR‐coated probe across a range of refractive index values. B) shift in localized surface plasmon resonance wavelength against the refractive index for a similar sensor using nanorods.

The sensor probe, featuring a CH film and a matrix of MUA‐capped GNRs, underwent exposure to diverse RI solutions to evaluate the influence of the CH film on the LSPR response. Variations in the solution's RI around the fiber‐optic sensor, coated with the nanoparticle matrix, can alter the LSPR characteristics, thereby inducing changes in absorbance. In our experimentation, glucose solutions with concentrations of between 20 and 90 mM in DI water were prepared, corresponding to an RI in the range of 1.373–1.395. Figure 6A illustrates the absorbance spectrum observed for the optical sensor probe modified with a CH‐coated fiber probe. The alterations in absorbance concerning different refractive indices are delineated in Figure 6B. Upon thorough analysis, the calculated average sensitivity for these variations was determined to be 502.69 Δ*ʎ*/RIU.

### 3.5 Absorbance measurements for oligomer alpha‐synuclein

. The proposed apparatus detects oligomer form of α‐syn by using CH. Given the GNRs’ sensitivity in detecting oligomeric α‐synuclein, the biosensing experiment was conducted at a stable RT of 25°C.

**FIGURE 7:**
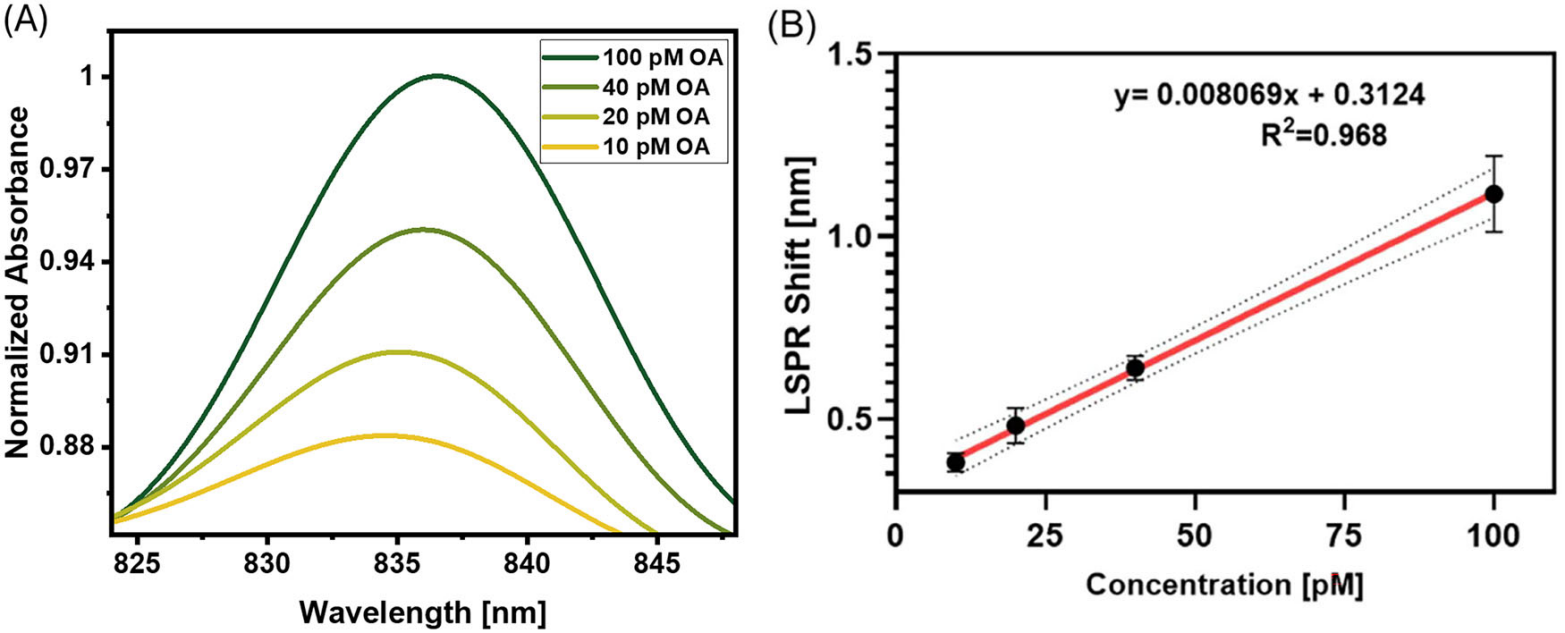
Absorbance values for oligomer alpha synuclein in PBS. (A) LSPR spectra of chitosan functionalized biosensor probe after treatment with PBS solutions having varying oligomer α‐syn concentrations, (B) Concentration versus LSPR shift plot. Curve fitting was performed using a linear fit equation on GraphPad Prism. The figure shows the best‐fit line (solid) within the confidence band (dashed).

Figure 7 illustrates the absorbance spectra corresponding to concentrations of 10, 20, 40, and 100 pM of oligomer α‐syn introduced into PBS. Notably, concentrations of oligomer α‐syn in the cerebrospinal fluid of PD patients typically range from 1 to 10 pM.^59^ Accordingly, the CH‐coated fiber optic probe was exposed to lower concentrations of OAs (10–100 pM). In Figure 7A, interestingly, lower oligomer concentrations demonstrated a proportional increase in the shift of peak absorption wavelength with rising OA concentration. However, a similar trend was not observed for absorbance values, identical to the detection of oligomer α‐syn protein in PBS. Specifically, upon binding 10 pM oligomer α‐syn, a redshift of  0.3 nm was detected, whereas a shift of  1.2 nm was observed with 100 pM oligomer α‐syn protein. In contrast, there was no observable wavelength shift in the control experiment using alpha‐synuclein‐free PBS. Analyzing the variation of peak absorbance wavelength shift against oligomer α‐syn concentration through regression analysis revealed linearity with an *R* ^2^ value of 0.968 (depicted in Figure 7B). The calculated LOD was determined to be 11 pM. The observed LOD is similar to that reported in a previous study using oligomer α‐syn protein^60^. Impressively, the proposed device enabled rapid diagnosis of α‐syn oligomer within 15 minutes, significantly quicker than conventional biosensors. This highlights the potential of the developed LSPR biosensor in PD diagnostics, with promising applications in point‐of‐care analyses.

### 3.6 Recovery, accuracy, and coefficient of variation analyses

Table 1 displays the accuracy/recovery and precision parameters (expressed as the coefficient of variation [CV%]) related to the concentration analysis of the oligomer form of α‐syn protein. The parameters were assessed based on 80%–120% accuracy/recovery conditions and <20% CV value.

**TABLE 1:** The values for accuracy/recovery and coefficient of variation (CV%) in the concentration analysis of oligomer alpha‐synuclein protein detection. The presented values include the calculated concentrations along with their respective standard deviations (SD).

Concentration (pM)	Calculated concentration (pM)	SD	Recovery %	CV%
10	9.7	1.0819	97.3	11.903
20	23.5	2.3119	118.65	9.838
40	42.33	0.4715	105.803	1.114
100	103	6.9761	103	6.773

*Note*: The presented values include the calculated concentrations along with their respective standard deviations (SD).

**FIGURE 8:**
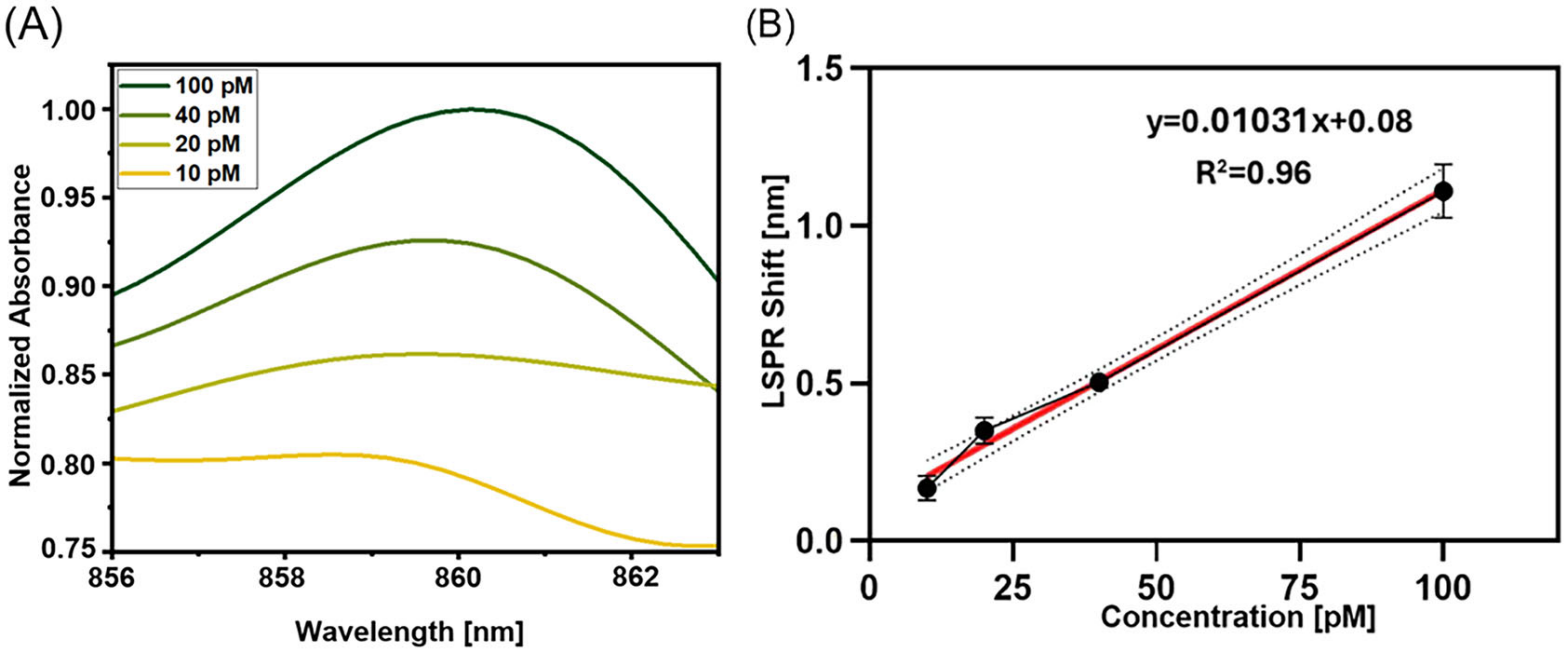
Absorbance values for oligomer alpha synuclein in Mimicked serum sample. (A) Absorbance spectra of chitosan functionalized biosensor probe after treatment with mimicked serum sample solutions having varying oligomer α‐syn concentrations, (B) Linearity plot of α‐syn oligomer fitted with linear parameter equation on GraphPad Prism from the output of absorbance spectra. Curve fitting was performed using a linear fit equation on GraphPad Prism. (OA: Oligomer alpha‐synuclein).

According to the results shown in Figure 8A,B, the oligomer α‐syn captured on the surface demonstrated good accuracy/recovery and low CV% values within the concentration range of 10–100 pM for oligomers α‐syn protein. The recovery rates for oligomer α‐syn proteins spiked with mimicked serum samples at concentrations of 10, 20, 40, and 100 pM using the proposed U‐shaped fiber optic biosensor were 97.30 %, 118.65 %, 105.83 %, and 103 %, respectively, with corresponding CV values of 11.903 %, 9.838 %, 1.114 %, and 6.773 %. This data confirms that the proposed sensing system is applicable for oligomer α‐syn protein with enough CV% and accuracy/recovery values in concentration analysis.

### 3.7 Selectivity of the sensing platform

**FIGURE 9:**
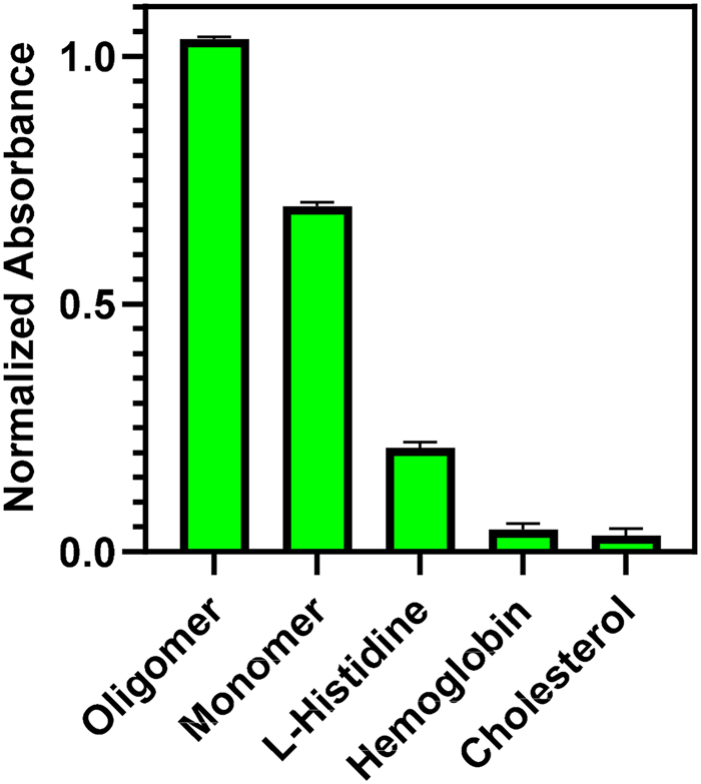
Selectivity test of the sensing system for oligomer form of α‐syn. The response values of cholesterol, hemoglobin, L‐histidine, monomer, and oligomer are separate. The error bars are standard deviations of three replicates.

CH is a polymer containing amino and hydroxyl groups, which enables the immobilization of various biomolecules and creates specific binding sites through these groups. The cholesterol, hemoglobin, l‐histidine, and monomer form of α‐syn protein samples were selected to verify the selectivity of the oligomer form of the alpha‐synuclein plasmonic sensor.Hemoglobin is found in high concentrations in biological samples;^61^ cholesterol was chosen to evaluate its potential interactions with lipids on the CH surface and cholesterol, in particular, is found in blood and is a critical component to consider in ensuring accurate detection in biological samples,^62^ and L‐histidine is a common amino acid found in the structure of proteins . It was chosen to see the interaction potential of the CH‐coated sensor surface with amino acids.^63^ In Figure 9, the response values of samples were obtained. The absorbance peak was lower than the oligomer alpha‐synuclein (100%) peak value for cholesterol (8.33%), hemoglobin (10%), and L‐Histidine (16.67%), and their response values were close. For alpha‐synuclein monomer, the selectivity percentage was found to be 66.33%. Alpha‐synuclein monomers and oligomers share structural similarities but also exhibit significant differences. Monomers are intrinsically disordered proteins, meaning they do not have a specific three‐dimensional structure and are highly flexible. Oligomers, on the other hand, are formed by the aggregation of monomers and typically exhibit more defined beta‐sheet structures, making them more stable. These structural characteristics can cause the sensor to respond closely to both forms.^64^ As shown in Figure 9, the oligomer α‐syn protein strongly interacted with the CH‐coated fiber probe, and no considerable shift was obtained in samples containing other interfering proteins. These results suggest that the sensor demonstrates good selectivity for oligomeric α‐synuclein, making it a promising tool for the detection of α‐synuclein oligomers, which are key markers in neurodegenerative diseases.

In this study, CH was successfully used as the biorecognition element. However, the close values of monomer and oligomer indicate a need for enhanced selectivity. As shown in Table  2, different LOD values obtained for the detection of alpha‐synuclein protein were reported. Future work could explore the use of specific antibodies or aptamers that are selective for oligomers together with CH to obtain a synergistic effect.^65^, ^66^ Such biorecognition elements could enable more effective recognition of oligomers, thereby further improving system performance

**TABLE 2:** Comparison of alpha‐synuclein detection reported in the literature.

Sensor	Method	Limit of detection (LOD)	Reference
Electrochemical biosensor	EIS	55 pM	^66^
Electrochemical biosensor	EIS	1.13 ng/mL	^67^
Electrochemical biosensor	CV	3.5 × 10^−5^ ng/mL	^68^
Electrochemical biosensor	SWV	0.03 fM	^69^
Electrochemical biosensor	CV	1 pM	^70^
Electrochemical biosensor	EIS	1.2 pM	^71^
Optical biosensor	Localized surface plasmon resonance (LSPR)	70 nM	^72^
Optical biosensor	Localized surface plasmon resonance (LSPR)	11 pM	This study

## 4 CONCLUSION

This study aims to design a highly sensitive and specific label‐free biosensor based on LSPR using GNRs, targeting a potential biomarker for PD. The biosensor was constructed by immobilizing GNRs onto a fiber probe and functionalizing the surface with appropriate molecules. In this investigation, the analyte of interest is an oligomeric form of alpha‐synuclein protein, which serves as a biomarker for PD. GNRs coated with MUA were employed to create a self‐assembled monolayer on the sensor surface. CH was conjugated onto the sensor surface to interact with the analyte. The RI sensitivity of the silanized GNR‐coated surfaces was determined to be 502.69 Δλ/RIU  nm/refractive index unit (RIU). The LOD in PBS was determined to be 11 pM. This study can be extended by exploring additional avenues, such as incorporating the system into a microfluidic platform to reduce sample volume and improve the control of the detection process.

## AUTHOR CONTRIBUTIONS

Begum Balkan Apaydın conceived, designed, and performed the experiments and collected the data. Tugay Çamoğlu, Begum Balkan Apaydın and Duygu Gezen‐Ak prepared and analyzed protein samples. Zeliha Cansu Canbek Özdil and Begum Balkan Apaydın prepared the gold nanorods and interpreted the data. Duygu Ege and Murat Gülsoy for review, editing, project administration, and funding acquisition.

## CONFLICT OF INTEREST STATEMENT

The authors declare no conflicts of interest.

## Supporting information

Supporting Information

## References

[1] Goedert M . Alpha‐synuclein and neurodegenerative diseases. Nat Rev Neurosci. 2001;2:492–501. 10.1038/35081564 11433374

[2] Tu W , Zheng C , Zheng Y , Feng Z , Lin H , Jiang Y , et al. The investigation of interaction and chaperon‐like activity of α‐synuclein as a protein in pathophysiology of Parkinson's disease upon direct interaction with tectorigenin. Int J Biol Macromol. 2023;249:125702. 10.1016/j.ijbiomac.2023.125702 37414324

[3] Hassanzadeh K , Morrone C , Akhtari K , Gerhardt E , Zaccagnini L , Outeiro TF , et al. Non‐SUMOylated alternative spliced isoforms of alpha‐synuclein are more aggregation‐prone and toxic. Mech Ageing Dev. 2023;209:3111759. 10.1016/j.mad.2022.111759 36464085

[4] Bendor JT , Logan TP , Edwards RH . The function of alpha‐synuclein. Neuron. 2013;79:1044–1066. 10.1016/j.neuron.2013.09.004 24050397 PMC3866954

[5] Singleton A , Farrer M , Johnson J , Singleton A , Hague S , Kachergus J , et al. α‐Synuclein locus triplication causes Parkinson's disease. Science. 2003;302:841. 10.1126/science.1090278 14593171

[6] Jo E , Fuller N , Rand RP , St George‐Hyslop P , Fraser PE . Defective membrane interactions of familial Parkinson's disease mutant A30P α‐synuclein. J Mol Biol. 2002;315:799–807. 10.1006/jmbi.2001.5269 11812148

[7] Spira PJ , Sharpe DM , Halliday G , Cavanagh J , Nicholson GA . Clinical and pathological features of a parkinsonian syndrome in a family with an Ala53Thr α‐synuclein mutation. Ann Neurol. 2001;49:313–319. 10.1002/ana.67 11261505

[8] Cuervo AM , Stefanis L , Fredenburg R , Lansbury PT , Sulzer D . Impaired degradation of mutant α‐synuclein by chaperone‐mediated autophagy. Science. 2004;305:1292–1295. 10.1126/science.1101738 15333840

[9] Burre J , Sharma M , Sudhof TC . Definition of a molecular pathway mediating alpha‐synuclein neurotoxicity. J Neurosci. 2015;35:5221–5232. 10.1523/JNEUROSCI.4650-14.2015 25834048 PMC4380997

[10] Gibb WR , Lees AJ . The relevance of the Lewy body to the pathogenesis of idiopathic Parkinson's disease. J Neurol Neurosurg Psychiatry. 1988;51:745–752. 10.1136/jnnp.51.6.745 2841426 PMC1033142

[11] Outeiro TF , Putcha P , Tetzlaff JE , Spoelgen R , Koker M , Carvalho F , et al. Formation of toxic oligomeric α‐synuclein species in living cells. PLoS One. 2008;3. 10.1371/annotation/9282f173-df82-4b70-9120-b4e62b3dacb1 PMC2270899 18382657

[12] Winner B , Jappelli R , Maji SK , Desplats PA , Boyer L , Aigner S , et al. In vivo demonstration that α‐synuclein oligomers are toxic. Proc Natl Acad Sci USA. 2011;108:4194–4199. 10.1073/pnas.1100976108 21325059 PMC3053976

[13] Cremades N , Cohen SI , Deas E , Abramov AY , Chen AY , Orte A , et al. Direct observation of the interconversion of normal and toxic forms of α‐synuclein. Cell. 2012;149:1048–1059. 10.1016/j.cell.2012.03.037 22632969 PMC3383996

[14] Lücking C , Brice A . Alpha‐synuclein and Parkinson's disease. Cell Mol Life Sci. 2000;57:1894–1908. 11215516 10.1007/PL00000671 PMC11146993

[15] Hsu LJ , Sagara Y , Arroyo A , Rockenstein E , Sisk A , Mallory M , et al. α‐synuclein promotes mitochondrial deficit and oxidative stress. Am J Pathol. 2000;157:401–410. 10934145 10.1016/s0002-9440(10)64553-1 PMC1850140

[16] Devi L , Raghavendran V , Prabhu B , Avadhani N , Anandatheerthavarada H . Mitochondrial import and accumulation of alpha‐synuclein impair complex I in human dopaminergic neuronal cultures and Parkinson disease brain. J Biol Chem. 2008;283:9089–9100. 18245082 10.1074/jbc.M710012200 PMC2431021

[17] Nakamura K , Nemani VM , Azarbal F , Skibinski G , Levy JM , Egami K , et al. Direct membrane association drives mitochondrial fission by the Parkinson disease‐associated protein alpha‐synuclein. J Biol Chem. 2011;286:20710–20726. 21489994 10.1074/jbc.M110.213538 PMC3121472

[18] Bourdenx M , Bezard E , Dehay B . Lysosomes and α‐synuclein form a dangerous duet leading to neuronal cell death. Front Neuroanat. 2014;8:83. 25177278 10.3389/fnana.2014.00083 PMC4132369

[19] O'Hara DM , Kalia SK , Kalia LV . Methods for detecting toxic α‐synuclein species as a biomarker for Parkinson's disease. Crit Rev Clin Lab Sci. 2020;57:291–307. 32116096 10.1080/10408363.2019.1711359

[20] Park MJ , Cheon SM , Bae HR , Kim SH , Kim JW . Elevated Levels of α‐Synuclein Oligomer in the Cerebrospinal Fluid of Drug‐Naïve Patients with Parkinson's Disease. J Clin Neurol. 2011;7:215. 22259618 10.3988/jcn.2011.7.4.215 PMC3259496

[21] Wang MJ , Yi S , Han JY , Park SY , Jang JW , Chun IK , et al. Oligomeric forms of amyloid‐β protein in plasma as a potential blood‐based biomarker for Alzheimer's disease. Alzheimer's Res Ther. 2017;9:98. 29246249 10.1186/s13195-017-0324-0 PMC5732503

[22] Fusco G , Chen SW , Williamson PTF , Cascella R , Perni M , Jarvis JA , et al. Structural basis of membrane disruption and cellular toxicity by a‐synuclein oligomers. Science. 2017;358:1440–1443. 29242346 10.1126/science.aan6160

[23] Kumar ST , Jagannath S , Francois C , Vanderstichele H , Stoops E , Lashuel HA . How specific are the conformation‐specific α‐synuclein antibodies? Characterization and validation of 16 α‐synuclein conformation‐specific antibodies using well‐characterized preparations of α‐synuclein monomers, fibrils and oligomers with distinct struct. Neurobiol Dis. 2020;146:105086. 32971232 10.1016/j.nbd.2020.105086

[24] Kelly SM , Jess TJ , Price NC . How to study proteins by circular dichroism. Biochim Biophys Acta. 2005;175(2):10,119–139. 10.1016/j.bbapap.2005.06.005 16027053

[25] Calero M , Gasset M . Fourier Transform Infrared and Circular Dichroism Spectroscopies for Amyloid Studies. Springer; 2005. pp. 129–151. 10.1385/1-59259-874-9:129 15980599

[26] Peng C , Gathagan RJ , Covell DJ , Medellin C , Stieber A , Robinson JL , et al. Cellular milieu imparts distinct pathological α‐synuclein strains in α‐synucleinopathies. Nature. 2018;557:558–563. 29743672 10.1038/s41586-018-0104-4 PMC5970994

[27] Lassen LB , Gregersen E , Isager AK , Betzer C , Kofoed RH , Jensen PH . ELISA method to detect α‐synuclein oligomers in cell and animal models. PLoS One. 2018;13(4):e0196056. 10.1371/journal.pone.0196056 29698510 PMC5919555

[28] De Giorgi F , Laferrière F , Zinghirino F , Faggiani E , Lends A , Bertoni M , et al. Novel self‐replicating α‐synuclein polymorphs that escape ThT monitoring can spontaneously emerge and acutely spread in neurons. Sci Adv. 2020;6(40). 10.1126/sciadv.abc4364 PMC7852382 33008896

[29] Pathak A , Shama P , Gupta BD . Ultrasensitive, highly selective and real‐time detection of protein using functionalized CNTs as MIP platform for FOSPR‐based biosensor. Nanotechnology. 2017, 10.1088/1361-6528/aa79e5 28617674

[30] Kumar R , Nguyen H , Rente B , Tan C , Sun T , Grattan KTV . A Portable 'Plug‐and‐Play' Fibre Optic Sensor for In‐Situ Measurements of pH Values for Microfluidic Applications. Micromachines. 2022;13(8):1224. 10.3390/mi13081224 36014146 PMC9416338

[31] Tan AJY , Ng SM , Stoddart PR , Chua HS . Theoretical Model and Design Considerations of U‐shaped Fiber Optic Sensors: a Review. IEEE Sensors J. 2020;20(24):14578–14589.

[32] Aydın EB , Aydın M , Sezgintürk MK . Highly selective and sensitive sandwich immunosensor platform modified with MUA‐capped GNPs for detection of spike Receptor Binding Domain protein: a precious marker of COVID 19 infection. Sens Actuators, B. 2021;345,130355. 10.1016/j.snb.2021.130355 PMC8225300 34188361

[33] Wang H , Ohnuki H , Endo H , Izumi M . Effects of self‐assembled monolayers on amperometric glucose biosensors based on an organic–inorganic hybrid system. Sens Actuators, B. 2012;168:249–255. 10.1016/j.snb.2012.04.018

[34] Vedhanayagam M , Andra S , Muthalagu M , Sreeram KJ . Influence of Functionalized Gold Nanorods on the Structure of Cytochrome –C: an Effective Bio‐nanoconjugate for Biomedical Applications. Inorg Chem Commun. 2022;146:110182. 10.1016/j.inoche.2022.110182

[35] Šimšíková M , Antalík M , Kaňuchová M , Škvarla J . Anionic 11‐mercaptoundecanoic acid capped ZnO nanoparticles. Appl Surf Sci. 2013;282:342–347. 10.1016/j.apsusc.2013.05.130

[36] Cao J , Galbraith EK , Sun T , Grattan KTV . Effective surface modification of gold nanorods for localized surface plasmon resonance‐based biosensors. Sens Actuators, B. 2012;169:360–367. 10.1016/j.snb.2012.05.019

[37] Al‐Mokaram A , Yahya R , Abdi MM , Mahmud HNME . The development of non‐enzymatic glucose biosensors based on electrochemically prepared polypyrrole‐chitosan‐titanium dioxide nanocomposite films. Nanomaterials. 2017;7:E119. 10.3390/nano7060129 PMC5485776 28561760

[38] Chikae M , Fukuda T , Kerman K , Idegami K , Miura Y , Tamiya E . Amyloid‐β detection with saccharide immobilized gold nanoparticle on carbon electrode. Bioelectrochemistry. 2008;74:118–123. 10.1016/j.bioelechem.2008.06.005 18676183

[39] Nangare S , Patil P . Chitosan mediated layer‐by‐layer assembly based graphene oxide decorated surface plasmon resonance biosensor for highly sensitive detection of β‐amyloid. Int J Biol Macromol. 2022;214:568–582. 10.1016/j.ijbiomac.2022.06.129 35752342

[40] Nikoobakht B , El‐Sayed MA . Preparation and Growth Mechanism of Gold Nanorods (NRs) Using Seed‐Mediated Growth Method. Chem Mater. 2003;15(10):1957–1962. 10.1021/cm020732l

[41] Ihse E , Yamakado H , van Wijk XM , Lawrence R , Esko JD , Masliah E . Cellular internalization of alpha‐synuclein aggregates by cell surface heparan sulfate depends on aggregate conformation and cell type. Sci Rep. 2017;7: 9008. 10.1038/s41598-017-08720-5 28827536 PMC5566500

[42] Sypabekova M , Amantayeva A , Gonzalez‐Vila A , Shaimerdenova M , Caucheteur C , Tosi D . Spectral characteristics and interrogation of a fiber‐optic ball resonator biosensor modulated by a tilted fiber Bragg grating. Opt Fiber Technol. 2023;79,103354.

[43] Kim HM , Bae SW , Park JH , Lee SK . Detection limit enhancement of fiber optic localized surface plasmon resonance biosensor by increased scattering efficiency and reduced background signal. Colloids Surf A. 2021;629:127439.

[44] Chyad RM , Jafri MZM , Ibrahim K . Fabricated nano‐fiber diameter as liquid concentration sensors. Results in Physics. 2013;3:91–96. 10.1016/j.rinp.2013.05.004

[45] Rodríguez‐Rodríguez WE , Puente‐Sujo JA , Rodríguez‐Rodríguez AJ , Matias IR , Vargas‐Requena DT , García‐Garza LA . Low‐Cost Online Monitoring System for the Etching Process in Fiber Optic Sensors by Computer Vision. Sensors. 2023;23:5951. 10.3390/s23135951 37447798 PMC10346387

[46] Rovera A , Tancau A , Boetti N , Dalla Vedova MDL , Maggiore P , Janner D . Fiber Optic Sensors for Harsh and High Radiation Environments in Aerospace Applications. Sensors. 2023;23:2512. 10.3390/s23052512 36904714 PMC10007412

[47] Ma S , Xu Y , Pang Y , Zhao X , Li Y , Qin Z , et al. Optical Fiber Sensors for High‐Temperature Monitoring: a Review. Sensors. 2022;22:5722. 10.3390/s22155722 35957279 PMC9371153

[48] del Caño R , Gisbert‐González JM , González‐Rodríguez J , Sánchez‐Obrero G , Madueño R , Blázquez M , et al. Effective replacement of cetyltrimethylammonium bromide (CTAB) by mercaptoalkanoic acids on gold nanorod (AuNR) surfaces in aqueous solutions. Nanoscale. 2020;12(2):658–668. 31829396 10.1039/c9nr09137h

[49] Shi X , Perry HL , Wilton‐Ely JDET . Strategies for the functionalisation of gold nanorods to reduce toxicity and aid clinical translation. Nanotheranostics. 2021;5(2):155–165. 10.7150/ntno.56432 33564615 PMC7868005

[50] Thierry B , Ng J , Krieg T , Griesser HJ . A robust procedure for the functionalization of gold nanorods and noble metal nanoparticles. Chem Commun. 2009, 1724–1726. 10.1039/B820137D 19294275

[51] Sahranavard M , Shahriari M , Abnous K , Hadizadeh F , Taghdisi SM , Zolfaghari R , et al. Design and synthesis of targeted star‐shaped micelle for guided delivery of camptothecin: in vitro and in vivo evaluation. Mater Sci Eng C. 2021;131:112529. 10.1016/j.msec.2021.112529 34857308

[52] Hasannia M , Abnou K , Taghdisi SM , Nekooei S , Ramezani M , Alibolandi M . Synthesis of doxorubicin‐loaded peptosomes hybridized with gold nanorod for targeted drug delivery and CT imaging of metastatic breast cancer. J Nanobiotechnology. 2022;20:391. 10.1186/s12951-022-01607-2 36045404 PMC9429417

[53] Jain PK , Eustis S , El‐Sayed MA . Plasmon Coupling in Nanorod Assemblies: Optical Absorption, Discrete Dipole Approximation Simulation, and Exciton‐Coupling Model. J Phys Chem B. 2006;110,37,18243–18253. 10.1021/jp063879z 16970442

[54] Cao J , Tu MH , Sun T , Grattan KTV . Wavelength‐based localized surface plasmon resonance optical fiber biosensor. Sens Actuators, B. 2013;181:611–619. 10.1016/j.snb.2013.02.052

[55] Sun Y , Yanagisawa M , Kunimoto M , Nakamura M . Depth profiling of APTES self‐assembled monolayers using surface‐enhanced confocal Raman microspectroscopy. Biomolecular Spectroscopy. 2017;184, 5,1–6. 10.1016/j.saa.2017.04.036 28475958

[56] Renger J . Excitation, Interaction, and Scattering of Localized and Propagating Surface Polaritons. Institut f¨ur Angewandte Physik, Dresden. Technische Universit¨at Dresden; 2006.

[57] Rafiee A , Mozafari N , Fekri N , Memarpour M , Azadi A . Preparation and characterization of a nanohydroxyapatite and sodium fluoride loaded chitosan‐based in situ forming gel for enamel biomineralization. Heliyon. 2024;10(2):e24217. 10.1016/j.heliyon.2024.e24217 38293392 PMC10825348

[58] Pacheco KML , Torres BBM , Sanfelice RC , da Costa MM , Assis L , Marques RB , et al. Chitosan and chitosan/turmeric‐based membranes for wound healing: production, characterization and application. Int J Biol Macromol. 2023;253(Part 7):127425. 10.1016/j.ijbiomac.2023.127425 37864933

[59] Hughes CD , Choi ML , Ryten M , Hopkins L , Drews A , Botía JA , et al. Picomolar concentrations of oligomeric alpha‐synuclein sensitize TLR4 to play an initiating role in Parkinson's disease pathogenesis. Acta Neuropathol. 2019;137(1):103–120. 10.1007/s00401-018-1907-y 30225556 PMC6338693

[60] Taghdisi SM , Danesh NM , Nameghi MA , Ramezani M , Alibolandi M , Hassanzadeh‐Khayat M , et al. A novel electrochemical aptasensor based on the nontarget‐induced high accumulation of methylene blue on the surface of electrode for sensing of α‐synuclein oligomer. Biosens Bioelectron. 2019;123:14–18. 10.1016/j.bios.2018.09.081 30278340

[61] Carlsson H , Rappaport SM , Törnqvist M . Protein Adductomics: methodologies for Untargeted Screening of Adducts to Serum Albumin and Hemoglobin in Human Blood Samples. High‐Throughput. 2019;8:6. 10.3390/ht8010006 30857166 PMC6473736

[62] Young MF , Oaks BM , Rogers HP , Tandon S , Martorell R , Dewey KG , et al. Maternal low and high hemoglobin concentrations and associations with adverse maternal and infant health outcomes: an updated global systematic review and meta‐analysis. BMC Pregnancy Childbirth. 2023;23:264. 10.1186/s12884-023-05489-6 37076797 PMC10114461

[63] Varlet‐Marie E , Ashenden M , Lasne F , Sicart MT , Marion B , de Ceaurriz J , et al. Detection of Hemoglobin‐Based Oxygen Carriers in Human Serum for Doping Analysis: confirmation by Size‐Exclusion HPLC. Clin Chem. 2004;50(4):723–731. 10.1373/clinchem.2003.026591 14764640

[64] Meade RM , Fairlie DP , Mason JM . Alpha‐synuclein structure and Parkinson's disease—lessons and emerging principles. Mol Neurodegeneration. 2019;14:29. 10.1186/s13024-019-0329-1 PMC6647174 31331359

[65] Jang SJ , Lee CS , Kim TH . α‐Synuclein Oligomer Detection with Aptamer Switch on Reduced Graphene Oxide Electrode. Nanomaterials. 2020;10:832. 10.3390/nano10050832 32349285 PMC7711764

[66] Bryan T , Luo X , Forsgren L , Morozova‐Roche LA , Davis JJ . The robust electrochemical detection of a Parkinson's disease marker in whole blood sera. Chem Sci. 2012, 3468. 10.1039/c2sc21221h

[67] Ge CY , Rahman MM , Zhang W , Lopa NS , Jin L , Yoon S , et al. An electrochemical immunosensor based on a self‐assembled monolayer modified electrode for label‐free detection of α‐synuclein. Sensors. 2020;20:617. 10.3390/s20030617 31979160 PMC7038178

[68] Ma Y , Qiong H , Liu C , Wang L . A nanospherical conjugated microporous polymer‐graphene nanosheets modified molecularly imprinted electrochemical sensor for high sensitivity detection of α‐Synuclein. J Electroanal Chem. 2020;862:1. 10.1016/j.jelechem.2020.113994

[69] Tao D , Gu Y , Song S , Nguyen EP , Cheng J , Yuan Q , et al. Ultrasensitive detection of alpha‐synuclein oligomer using a PolyD‐glucosamine/gold nanoparticle/carbon‐based nanomaterials modified electrochemical immunosensor in human plasma. Microchem J. 2020;158. 10.1016/j.microc.2020.105195

[70] Sun K , Xia N , Zhao L , Liu K , Hou W , Liu L . Aptasensors for the selective detection of alpha‐synuclein oligomer by colorimetry, surface plasmon resonance, and electrochemical impedance spectroscopy. Sens Actuator B: Chem. 2017;245:87–94. 10.1016/j.snb.2017.01.171

[71] Xu Q , Cheng H , Lehr J , Patil AV , Davis JJ . Graphene oxide interfaces in serum‐based autoantibody quantification. Anal Chem. 2015;87:346–350. 10.1021/ac503890e 25514013

[72] Khatri A , Punjabi N , Ghosh D , Maji SK , Mukherji S . Detection and differentiation of α‐Synuclein monomer and fibril by chitosan film coated nanogold array on optical sensor platform. Sens Actuators B: Chem. 2018;255:692–700. 10.1016/j.snb.2017.08.051

