# Supplementary figures and images for: Chitosan‐enhanced sensitivity of mercaptoundecanoic acid (MUA)‐ capped gold nanorod based localized surface plasmon resonance (LSPR) biosensor for detection of alpha‐synuclein oligomer biomarker in parkinson’s disease
Source: Biotechnol Appl Biochem. 2024 Sep 3;72(1):150–63. doi: 10.1002/bab.2653 (PMC11798540; doi:10.1002/bab.2653)

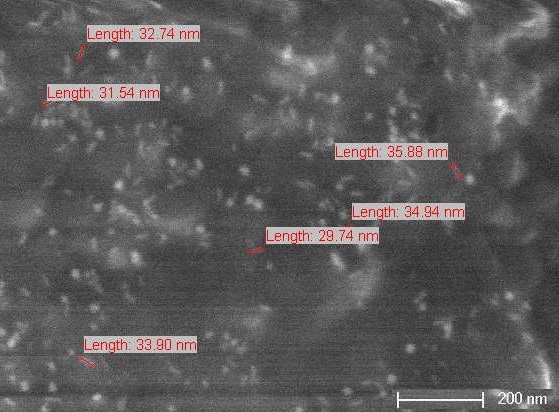


**Figure S1.** SEM images for the MUA-capped Gold Nanorods size

Supplement: Supplementary file 1 — Supporting Information [file BAB-72-150-s001.docx]
